# Building a database for curriculum mapping and analytics

**DOI:** 10.15694/mep.2019.000038.1

**Published:** 2019-03-04

**Authors:** Yew-Beng Kang, Vishna Devi D. Nadarajah

**Affiliations:** 1 International Medical University

**Keywords:** Curriculum map, analytics, building a curriculum database, search

## Abstract

This article was migrated. The article was marked as recommended.

Curriculum mapping of an outcomes-based programme using a database was developed using a relational database structure, and it functions as a searchable database by the use of keywords. Factors such as the framework of the programme, the database entity relationship diagram (ERD), benchmarking, terminology and nomenclature, analytics and integration with the learning management system requires careful consideration before implementation. Built into the structure of the curriculum database are the analytics features which identifies the curricula data using defined keywords. This enables staff and students to search through any programme or subject of interest to track a subject or keyword to the point of delivery with the use of the analytics feature. This results in the curricula information being transparent for all stakeholders, ensuring curriculum mapping and blueprinting of assessments are readily available.

This paper reports on the implementation of a university-wide curricula database which includes multiple undergraduate and postgraduate programmes, including chronological versions of a programme at an institution with diverse health professions programmes, including medicine, dentistry, pharmacy. Additionally, this paper outlines the steps to design the curricula database, the development of the framework of the database and the analytics, the challenges in implementation, the results that can be obtained from such a database and the lessons learnt.

## Introduction

Outcomes-based education (OBE) is a way of “designing, developing, delivering, and documenting instruction in terms of its intended goals and outcomes” ( Spady, 1988). This performance-based approach to curriculum development coupled with constructive alignment between the curriculum, delivery and assessments, necessitates a requirement to track, map, and identify the evidence of a particular lesson that has been planned, delivered and assessed in the curriculum ( Biggs, 1996). Challenges in tracking the curriculum have suggested an urgency in developing a tool that allowed the stakeholders to access information for an OBE curriculum ( Harden, 2001).

Many tools have been proposed to support curriculum mapping. One of the first reported databases for a medical curriculum came about in 1997 and was from the College of Medicine at the University of Iowa ( University of Iowa, Health Care, Medical Curriculum Repository, 2017). This database was organised by course and the search was done by subject and keywords. A curriculum database is generally regarded as an electronic repository of a curriculum ( Mattern *et al.*, 1992). The database is an essential tool to organise large amounts of data, especially a curriculum, which contains information on outcomes, objectives, teaching and learning activities, credit hours, themes, topics and student learning time, amongst the many elements in the OBE curriculum ( Friedman, 1995). Another widely used curriculum database in North American medical schools is the CurrMIT ( Salas *et al.*, 2003).

Currently, there are many software applications that can be used for curriculum mapping, and they are in the form of a spreadsheet, or a database (Curriculum 21, 2009-2017; Entrada Consortium, 2017; Liftupp, 2017). Databases are the preferred solution for curriculum mapping as they are capable of organising data into fields and records and are designed for data management ( Masters, 2018). These curriculum mapping software applications are usually integrated with other student learning tracking capabilities such as portfolios, timetable scheduling, learning management system, and assessment tracking as well as student feedback. These features are built-into the mapping software and may not be configurable or may not be what the end user needs. Curriculum mapping must meet the needs of administrators, faculty and students as well as support the needs of initiatives such as continuous quality improvement, curriculum renewal, accreditation, support of curriculum committees, reports to accreditation committees, and medical education research. In light of these requirements, we have built a curriculum map based on these requirements and needs at our institution which offers diverse health professions programmes at both undergraduate and postgraduate levels.

## Methods

Although there are many commercially available solutions to support curriculum mapping, a purpose-built application was identified to be more useful for our institution, the International Medical University in Kuala Lumpur. There were several factors to consider.

**Figure 1.**
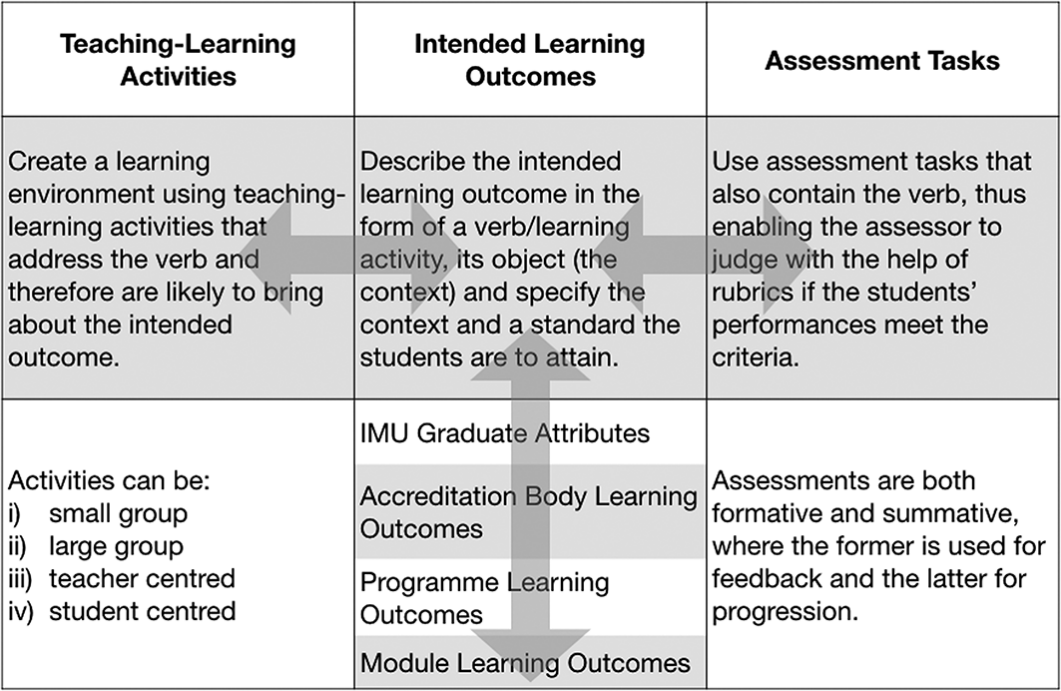
Constructive alignment framework used for the framework of the curriculum map

### Phase 1: Planning the Curriculum Database Structure

The following decisions were considered first as they affected the entity relationship diagram (ERD) of the database, which was crucial to the curriculum alignment framework built to support the search and analysis functions ( Biggs, 1996) ( Figure 1). User needs obtained from curriculum administrators and faculty members were included in the decisions. The seven decisions made at International Medical University were as follows:

1. Framework of the curriculum structure: A constructive alignment framework was used, where the intended learning outcomes were aligned with the teaching/learning activities and the assessments.
2. Database Type: A relational database structure was used to enable both vertical and horizontal integration of the curriculum which allows for searching capabilities using the constructive alignment framework.
3. Requirements for Benchmarking: Benchmarking of the outcomes with accreditation bodies standards.
4. Define terminology and nomenclature: A standardised terminology and use of terms throughout the curriculum database was established.
5. Analytics functionality: Ability to perform data analytics to analyse curricula details and display in a graphical form, including tracking and dashboarding.
6. Capacity for Integration: Enable links to the current learning management system and the ability to import and export data.
7. User training and support documentation: Periodic user training and online supportdocumentation was provided to ensure that users are supported.

**Figure 2.**
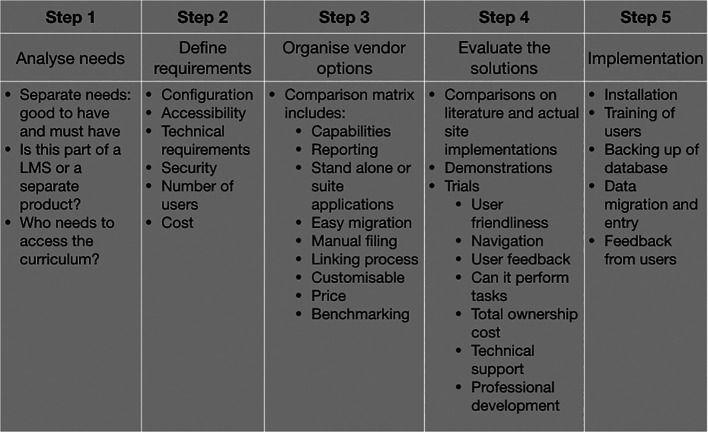
Steps required to develop a curriculum database

#### Database Application Development

The following decisions were used as a guide to develop the database application based on the following steps ( Perez, 2016) ( Figure 2). These are:

Step 1: Analyse needs and separate the ‘must haves’ from the ‘good to have’ for the curriculum database and identify the various stakeholders.

Step 2: Define requirements for technical needs to support the database application for the required number of users.

Step 3: Organise the database application vendor comparison matrix to select the appropriate vendor/solution based on requirements.

Step 4: Evaluate the different solutions from the application vendors based on technical feedback, demonstrations and user trials, and selection of appropriate solution.

Step 5: Implementation of the database including installation, training, of users, backup of the database and feedback for process improvement.

### Phase 2: Development

The curriculum map developed with the above considerations can be used to track individual programmes and courses/modules as well as individual lessons, and can also track multiple programmes including different versions of the same programme ( Kang, 2012). At the IMU, the curriculum map is known as the curricula database and it involves all the undergraduate programmes (12) in the university and tracks 109 separate database fields, of which 58 of them are curricula descriptors. The database was also contextualised to the IMU’s and the Malaysian Accreditation Board requirements such as the Code of Practice for Programme Accreditation (COPPA) for the cognitive, affective and psychomotor domains and soft skills (Malaysian Qualifications Agency, 2007).

It is capable of tracking any keyword, or term and map it to outcomes, teaching learning methods, curricula content and assessment tools. Other than blueprinting assessment tools to learning outcomes, the database allows for blueprinting of Malaysian Qualification Agency accreditation COPPA domains to components in the curricula for each programme. Reference materials including books, websites and e-learning links can also be map and tracked across the various programmes.

**Table 1.** Example of curricula mapping results obtained with analytics.

	Using Data from	Search Through/For	Analytics applied	Example
1	Programme/Semester/Module	Learning Activities	Sort the data into delivery types encompassing soft skills, cognitive, affective and psychomotor domains	Searching for where ophthalmology is taught in Medicine and how the various domains are used to achieve the outcomes.
2	Outcome domains	Undergraduate programmes	Identifying which programmes or modules covers entrepreneurship as a subject	Searching for where “Entrepreneurship” is delivered for any programme in the university and if inter-professional learning activities are possible.
3	Programme/Semester/Module	Accreditation requirements in soft skills domains	Sorting the delivery by module, semester or delivery type	Searching through the Nursing programme to identify where “thinking outside the box” takes place in the programme
4	Assessment blueprint	Tools used for summative assessment	Sort the data into programmes, modules or semesters which then can identify support of reflective writing for the students and staff	Searching for reflection as a summative assessment in the curriculum and identifying where teaching and learning resources are needed to support both staff and students.
5	Support material and references recommended for student use	Usage by different programmes	Analysis of the resources used can help the chief librarian to manage the inventories.	Searching for textbooks by a certain author or title and if it is used in any programme.
6	Programme outcomes	Mapping various outcomes from different programmes to the accreditation body outcome domains	Analysis of the delivery at particular time points	Identification of potential of inter-professional learning across different cohorts to achieve similar outcomes.

### Phase 3: Database Mining and Graphical Output

**Figure 3.**
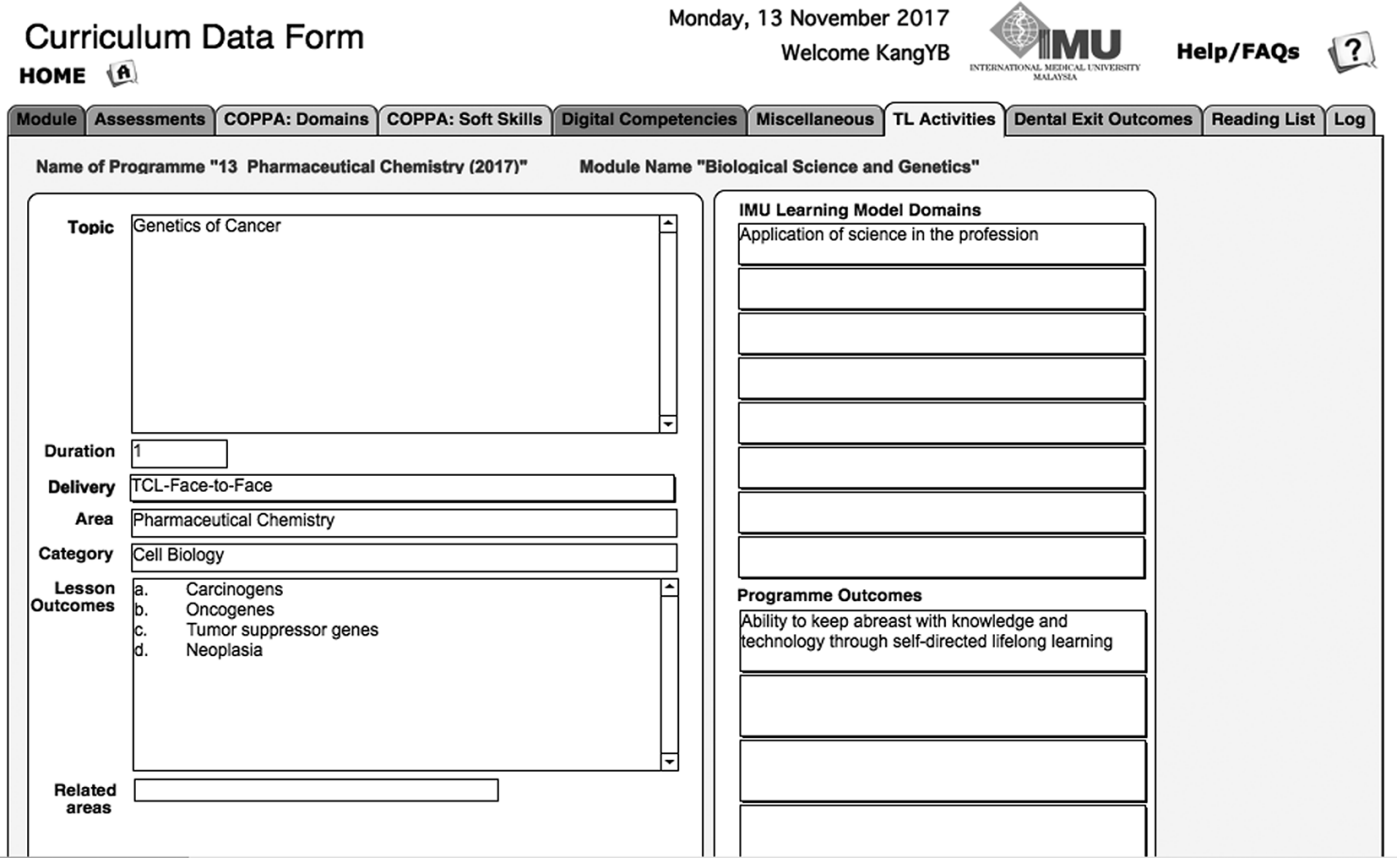
Example of mapping in the curriculum database between a lesson/topic with the learning domains and programme outcomes

The curricula database has embedded analytics functions where the search results can be analysed and sorted using any function or fields to give comprehensive details ( Table 1). It addresses the issues of making the curricula transparent to the students, faculty and administrators. It can generate reports for mapping of the curricula to outcome descriptors and this leverages on the capabilities of a relational database. This database, enabled individual tracking of fields within a programme as well as the ability to track keywords or topics which may be taught across programmes (eg. identifying how ethics and professionalism is delivered by different programmes). Such mapping helps staff and administrators during the accreditation and curriculum review processes, and since access of curricula map is through the cloud, everyone in the university including students can view the map. Additionally, the relational structure of the database allows staff from different programmes to look for opportunities to conduct common learning as well as inter-professional learning, thus maximising the use of scarce resources. Figure 3 illustrates an example where the database was mined to show a mapping between a lesson/topic with learning domains and programme outcomes.

#### The challenges faced:

(1) Structure of the database: The structure of the database is an important decision as the wrong structure will result in the inability for the database to search, analyse and generate reports and graphical analysis. It is recommended that in developing the database structure and the entity relationship diagram (ERD), that personnel who are fluent in developing database structures are involved and are included in part of the team.

(2) Faculty and staff buy-in: A curriculum map is a living document and will need to be updated on a periodic basis to reflect the currency of the curriculum. These are challenges for faculty and the curriculum administrator. One solution to solve this issue is to provide Word/Excel templates with the curriculum details so as to aid the automatic import of the information into the database. Currency of the curriculum data can be tracked by four fields in the database which identifies the person who enters the data, the person who modifies the data,was modified. This information is supported with a log file which contains the history of changes.

(3) Standardised terminology and nomenclature: This is an important issue to consider, especially when multiple programmes from different disciplines are using the curriculum database. A common example is the use of the term lecture and plenary interchangeably. To resolve this issue, an extensive frequently asked questions (FAQ) section on the database includes the list of terminology accepted and used in the database.

## Lessons Learnt

The process of developing a bespoke curriculum map using a database is an involved, iterative, and consultative process. The process of mapping should be started with a single programme, preferably one with many common educational elements across the university. One alternatively can construct the database one each for individual programmes, however, such a decision does not exploit the capabilities of the curriculum database to map through the entire curricula.

Other considerations are the accessibility and security access for users. Depending on the security and privacy level of the university and policies, the access to the database may be limited to users accessing it from specific IP addresses (campus limited). The curricula map data generally belongs to the university and there must be processes for those accessing it. Privileges for those who are viewing the data must be different from those who are updating or involved in data entry to distinguish between the two tasks and to prevent accidental deletion and modification. There should be a built-in log file to track what are the changes made and the individuals who have made the changes it. Another important matter is the how the curriculum map would look for the user on the computer and on mobile devices. The screen real estate for these devices are different and the application should dynamically recognise the access of such devices and display appropriate information on demand. Finally, the curricula database project should be self-sustaining, that is if the programmer/developer is no longer in service, the database can be run with less human intervention.

## Moving Forward

Curriculum mapping database with built-in analytics is an important tool to assist with curriculum management, resource tracking, and curriculum reviewprocesses. Ultimately, curriculum mapping is used to assist teaching and learning as well as preparation of learning plans by the students. Integration between the database with the learning management system (LMS) can enable the learners to be responsible for their own learning plans. For curriculum and university administrators, the database can be modified to provide a dashboard to provide a macro view of the curriculum for resource planning and administration.

Furthermore, integration of artificial intelligence (AI) to track student usage of the database can provide insight into the user engagement which can but help improve the outcomes on how the map can be used to improve outcomes.

## Conclusion

The curricula database is useful and powerful tool in mapping, tracking and identifying curricula data and events. This becomes even more powerful with the addition of analytics, which enables the sorting of the queries.

## Take Home Messages

• Planning the Curriculum Database Structure
• Database Application Development
• Development of the Application
• Database Mining and Graphical Output

## Notes On Contributors

Yew-Beng Kang is an Associate Professor and the Associate Dean E-Learning, International Medical University, Kuala Lumpur, Malaysia. ORCHID: https://orcid.org/0000-0001-6733-8880

Vishna Devi D. Nadarajah is a Professor and the Pro Vice-Chancellor of Education, International Medical University, Kuala Lumpur, Malaysia.
