# Building a database for curriculum mapping and analytics

**DOI:** 10.15694/mep.2019.000038.2

**Published:** 2019-08-19

**Authors:** Yew-Beng Kang, Vishna Devi D. Nadarajah, Vishna Devi V. Nadarajah

**Affiliations:** 1 International Medical University

**Keywords:** Curriculum map, analytics, building a curriculum database, search

## Abstract

This article was migrated. The article was marked as recommended.

Curriculum mapping of an outcomes-based programme using a database was developed using a relational database structure, and it functions as a searchable database by the use of keywords. Factors such as the framework of the programme, the database entity relationship diagram (ERD), benchmarking, terminology and nomenclature, analytics and integration with the learning management system requires careful consideration before implementation. Built into the structure of the curriculum database are the analytics features which identifies the curricula data using defined keywords. This enables staff and students to search through any programme or subject of interest to track a subject or keyword to the point of delivery with the use of the analytics feature. This results in the curricula information being transparent for all stakeholders, ensuring curriculum mapping and blueprinting of assessments are readily available.

This paper reports on the implementation of a university-wide curricula database which includes multiple undergraduate and postgraduate programmes, including chronological versions of a programme at an institution with diverse health professions programmes, including medicine, dentistry, pharmacy. Additionally, this paper outlines the steps to design the curricula database, the development of the framework of the database and the analytics, the challenges in implementation, the results that can be obtained from such a database and the lessons learnt.

## Introduction

Outcomes-based education (OBE) is a way of “designing, developing, delivering, and documenting instruction in terms of its intended goals and outcomes” ( Spady, 1988). This performance-based approach to curriculum development coupled with constructive alignment between the curriculum, delivery and assessments, necessitates a requirement to track, map, and identify the evidence of a particular lesson that has been planned, delivered and assessed in the curriculum ( Biggs, 1996). Challenges in tracking the curriculum have suggested an urgency in developing a tool that allowed the stakeholders to access information for an OBE curriculum ( Harden, 2001).

Many tools have been proposed to support curriculum mapping. One of the first reported databases for a medical curriculum came about in 1997 and was from the College of Medicine at the University of Iowa (University of Iowa, Health Care, Medical Curriculum Repository, 2017). This database was organised by course and the search was done by subject and keywords. A curriculum database is generally regarded as an electronic repository of a curriculum ( Mattern *et al.*, 1992). The database is an essential tool to organise large amounts of data, especially a curriculum, which contains information on outcomes, objectives, teaching and learning activities, credit hours, themes, topics and student learning time, amongst the many elements in the OBE curriculum ( Friedman, 1995). Another widely used curriculum database in North American medical schools is the CurrMIT ( Salas *et al.*, 2003).

Currently, there are many software applications that can be used for curriculum mapping, and they are in the form of a spreadsheet, or a database (Curriculum 21, 2009-2017; Entrada Consortium, 2017; Liftupp, 2017). Databases are the preferred solution for curriculum mapping as they are capable of organising data into fields and records and are designed for data management ( Masters, 2018). These curriculum mapping software applications are usually integrated with other student learning tracking capabilities such as portfolios, timetable scheduling, learning management system, and assessment tracking as well as student feedback. These features are built into the mapping software and may not be configurable or may not be what the end user needs. Curriculum mapping must meet the needs of administrators, faculty and students as well as support the needs of initiatives such as continuous quality improvement, curriculum renewal, accreditation, support of curriculum committees, reports to accreditation committees, and medical education research. In light of these requirements, we have built a curriculum map based on these requirements and needs at our institution which offers diverse health professions programmes at both undergraduate and postgraduate levels.

## Methods

Although there are many commercially available solutions to support curriculum mapping, a purpose-built application was identified to be more useful for our institution, the International Medical University (IMU) in Kuala Lumpur.

### Phase 1: Planning the Curriculum Database Structure

The following decisions were considered first as they affected the entity relationship diagram (ERD) of the database, which was crucial to the curriculum alignment framework built to support the search and analysis functions ( Biggs, 1996) ( Table 1). User needs obtained from curriculum administrators and faculty members were included in the decisions. The seven decisions made at International Medical University were as follows:

• Framework of the curriculum structure: A constructive alignment framework was used, where the intended learning outcomes were aligned with the teaching/learning activities and the assessments.
• Database Type: A relational database structure was used to enable both vertical and horizontal integration of the curriculum which allows for searching capabilities using the constructive alignment framework.
• Requirements for Benchmarking: Benchmarking of the outcomes with accreditation bodies standards.
• Define terminology and nomenclature: A standardised terminology and use of terms throughout the curriculum database was established.
• Analytics functionality: Ability to perform data analytics to analyse curricula details and display in a graphical form, including tracking and dashboarding.
• Capacity for Integration: Enable links to the current learning management system and the ability to import and export data.
• User training and support documentation: Periodic user training and online support documentation was provided to ensure that users are supported

**Table 1.** Constructive alignment framework used for the framework of the curriculum map

Teaching-Learning Activities	Intended Learning Outcomes	Assessment Tasks
Create a learning environment using teaching-learning activities that address the verb and therefore are likely to bring about the intended outcome.	Describe the intended learning outcome in the form of a verb/learning activity, its object (the context) and specify the context and a standard the students are to attain.	Use assessment tasks that also contain the verb, thus enabling the assessor to judge with the help of rubrics if the students’ performances meet the criteria.
Activities can be: 1. Small group 2. Large group 3. Teacher-centred 4. Student-centred	University Graduate Attributes Accreditation Body Learning Outcomes Programme Learning Outcomes Module Learning Outcomes	Assessments are both formative and summative, where the former is used for feedback and latter for progression.

#### The IMU experience:

The first four decisions (above) resulted in a consistent relational database design. As a result, the database was easier to perform analytics on as the relational database structure was able to link the outcomes, delivery and assessments in the curriculum map/database. Such mapping can be achieved using a spreadsheet, but spreadsheets are essentially a two-dimensional grid where the rows and the columns of the spreadsheet are linked together in table. Curriculum maps generally correlate far more complex relationships and for the example of constructive alignment for Course Objectives to Teaching and Learning to Assessments; there should be at least three tables to link all the three relationships. Such spreadsheets will become exceedingly complex as there are many numerous Course Objectives, Teaching and Learning activities and Assessments for a single course. Imagine the spreadsheet being used for a programme with multiple courses over 8 semesters (or even more); the spreadsheet requirements can be complex with multiple use of rows and columns to map the relationships.

In this case, the database is the preferred solution as it is a collection of related tables (like in a spreadsheet) which is linked together in an entity relationship diagram (ERD). A database can manage large amounts of data and the relational database allows one point of change for data that share the same information. For example, programmes that share similar institutional outcomes, if there is an institutional or accreditation need to change the outcome that is shared by these programmes, there need only to change it once from the database point of view. Similarly, there are many shared descriptors or elements for the curriculum, in which the one point of change for the relational database will result in less errors, mishaps and omissions in the tracking of changes to the curriculum since duplication is kept at a minimum.

**Figure 1.**
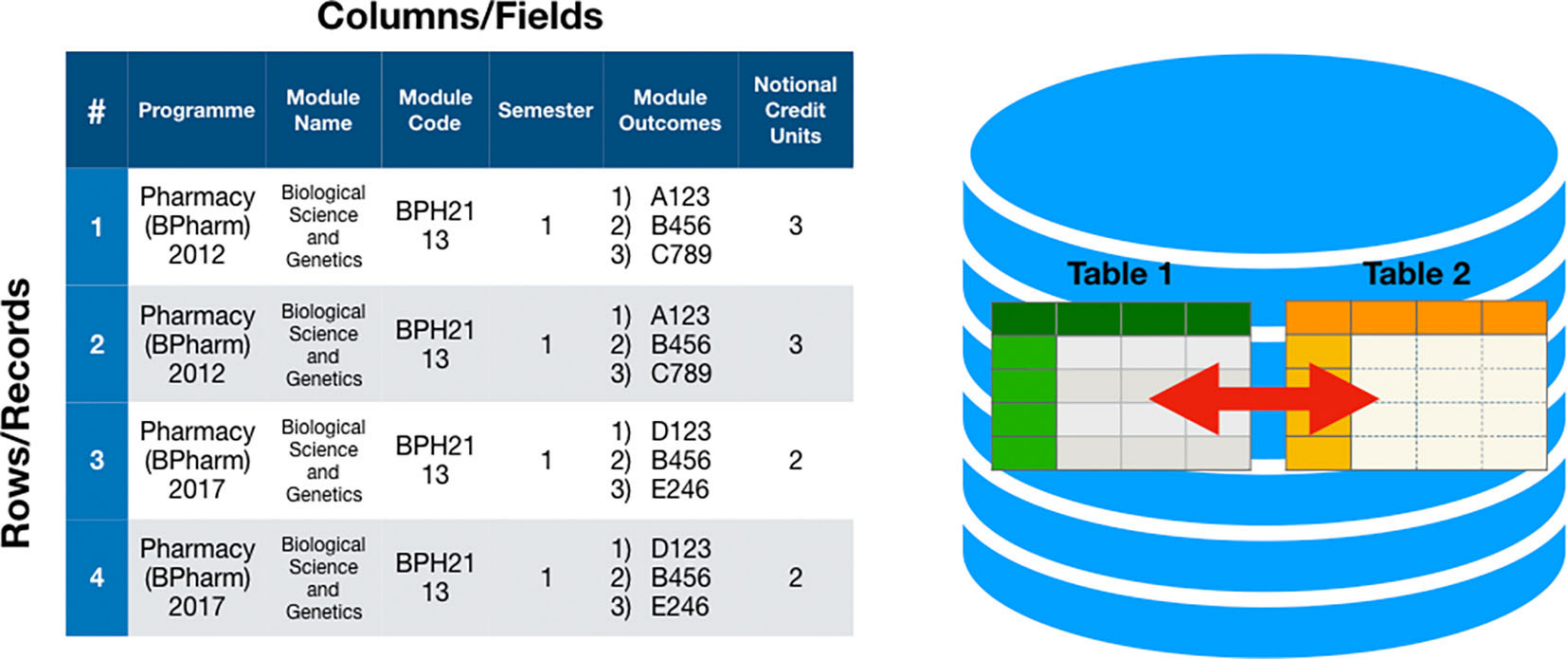
Relationship between rows and columns of a spreadsheet with a set of tables in a database

Data modelling of the curriculum requires a thoughtful process of organising the curriculum into entities (tables) and attributes (columns), which is the database terminology for what is normally tables and columns in the spreadsheet ( Figure 1). Databases are a collection of records or data and there are currently several ways of managing these data - and the database system is usually named after the way it manages the data.

Curriculum data for any educational programme following an outcomes-based objective typically can be separated into the objectives/outcomes, delivery of the teaching and learning and the assessment. There is an inherent relationship between these three areas as established in constructive alignment and changes in any one of the areas will impact the other two. In other words, the curriculum structure for an outcomes-based programme should best be constructed as a relational database where separate tables of data are linked with one another through a relationship. In Figure 1 the table on the left lists the rows and columns of a spreadsheet or the records and fields of a table in a database. Tables are all inter-related with a defined relationship so that table 1 can refer to information on table 2 and vice versa. Multiple types of data including updated revisions of data in the curriculum can be maintained in the tables: The table on the left illustrates old data in rows 1 and 2, whereas rows 3 and 4 are the updated curriculum data and they reside on the same database table.

#### Database Application Development:

The steps on developing the application was based on the steps listed in Table 2 ( Perez, 2016). These steps include an analysis of the needs and establishing project management approach. The steps are listed in Table 2 are self-explanatory and identify the questions the project team needs to ask themselves.

**Table 2.** Steps required to develop a curriculum database ( Perez, 2016)

*Step 1*	*Step 2*	*Step 3*	*Step 4*	*Step 5*
*Analyse needs*	*Define requirements*	*Organise vendor options*	*Evaluate the solutions*	*Implementation*
• Separate needs: good to have and must have • Is this part of a LMS or a separate product? • Who needs to access the curriculum?	• Configuration Accessibility • Technical requirements • Security • Number of users • Cost	• Comparison matrix includes: • Capabilities • Reporting • Stand alone or suite applications • Easy migration • Manual filing • Linking process • Customisable • Price Benchmarking	• Comparisons on literature and actual site implementations • Demonstrations • Trials • User friendliness • Navigation • User feedback • Can it perform tasks • Total ownership cost • Technical support • Professional development	• Installation • Training of users • Backing up of database • Data migration and entry • Feedback from users

#### The IMU experience:

All the five steps listed in Table 2 were considered, albeit not necessarily in the sequential order listed here. The needs analysis and coverage of requirements will normally require the input of IT literate faculty or better still Information Technology Services department or developers. At this stage, one must be very clear for what the goals of the database is for. This can be a confusing time as there are goals which are must haves and those which are good-to-have and usually they differ between different users of the database. The accreditation needs will usually be different from the teaching faculty’s needs as well as for the students. Having a very clear idea of the goals will assist this step. In our experience, we considered the needs of everyone who will have access to the database: namely the university quality processes (accreditation), the teaching and learning delivery team (faculty), and the users (students). These categories will have an impact on the user rights in accessing the database.

Historically, the database began its life mapping a single programme and had evolved its various database tables, relationships, curriculum descriptors and functionalities for the BSc. Pharmacy (Hons) with one of the authors as the developer. The functions and needs of the database grew ‘organically’ as the needs of the programme grew and only was the opportunity to include the curricula from other progammes was taken. Even though the curricula database was an in-house development, the steps listed under Table 2 was necessary at each stage of its growth.

### Phase 2: Development

#### Setting Ground Rules

**Figure 2.**
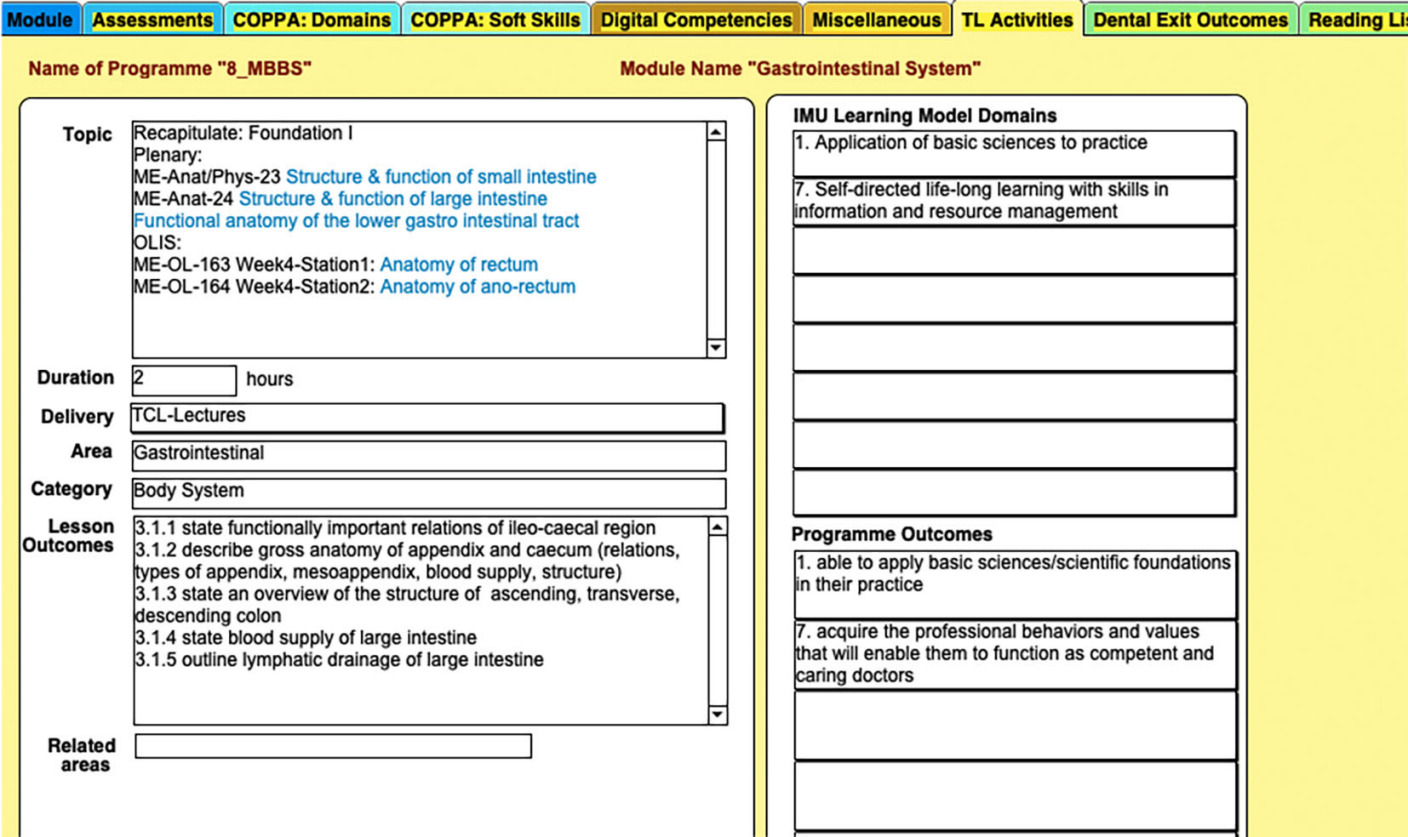
Example of mapping in the curriculum database between a lesson/topic with the learning domains and programme outcomes

**Figure 3.**
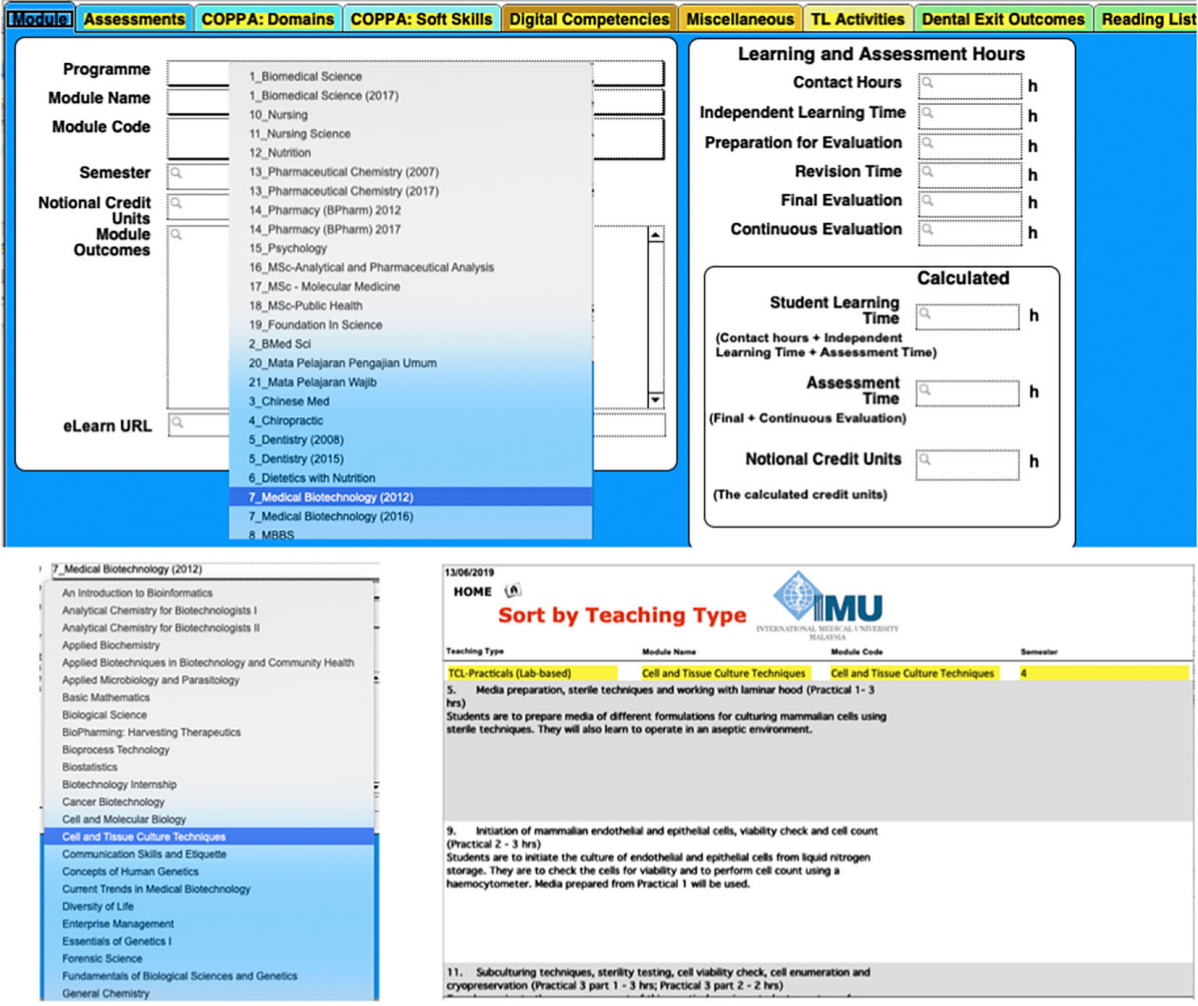
Example on selection of a programme from list (top), followed by related modules (below left) and analytics that sorts the data according to types of delivery (below right)

**Figure 4.**
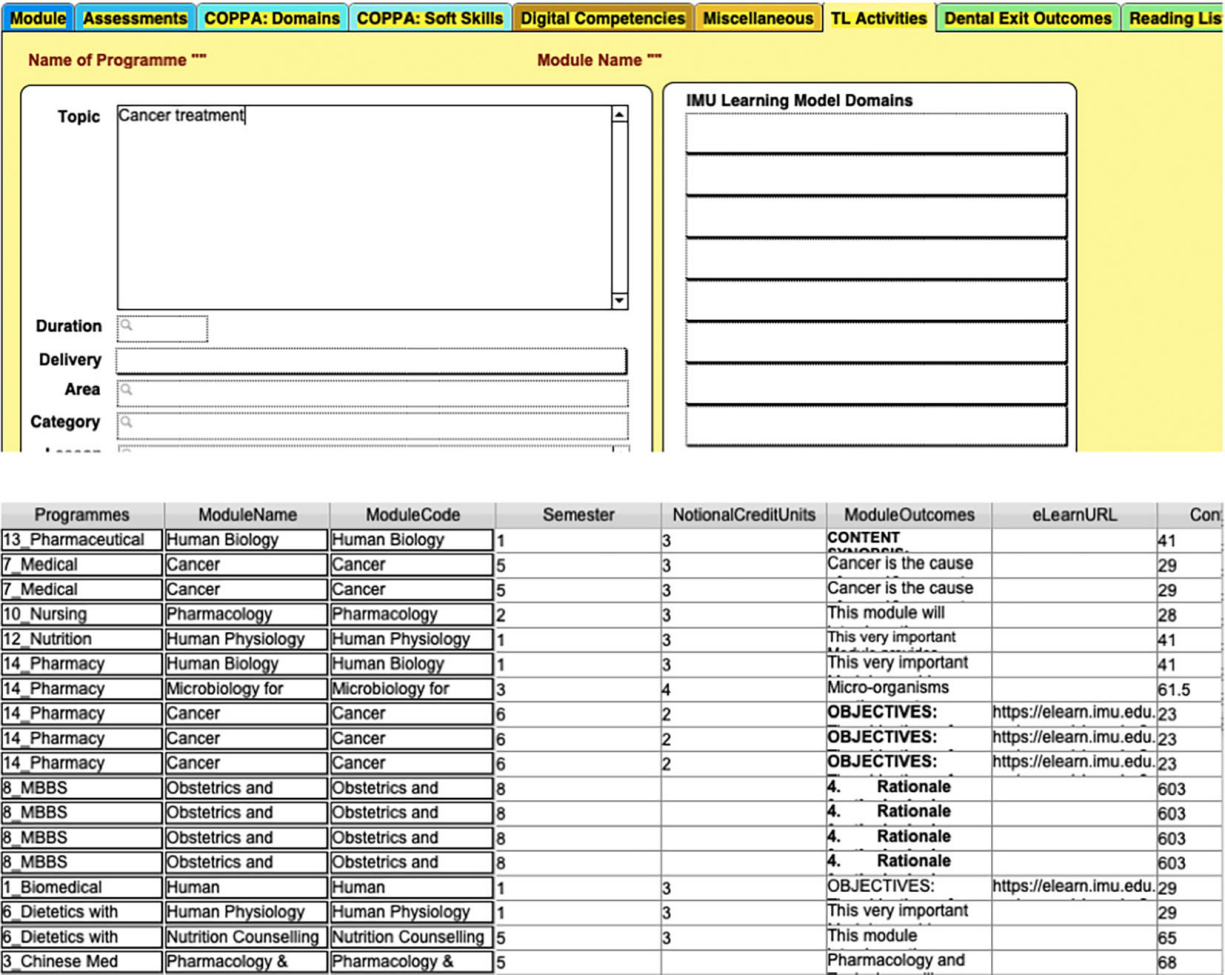
An example of a search of key words from the topic descriptor (top) leading to a list of results (bottom)

Commercial curriculum mapping software have standardised name fields which may be difficult if not impossible to change, and during the initial planning phase for the project, the five ground rules set were:

• Common curriculum descriptors were to be used for all programmes and systems,
• Unique curriculum descriptors for a minority number of programmes may be used if necessary,
• The smallest unit of measure for any programme is the teaching and learning delivery (the curricula database is a collection of teaching and learning activities),
• All items under (3) can be mapped in the architecture of the curriculum and nested under modules, programmes, assessments, accreditation domains, competencies, teaching and learning activities, outcomes, and references/reading list,
• A search through any of the curriculum descriptors in (4) can derive any of the other linked descriptors (See Figures 2, 3, 4)

With the above ground rules, there were several factors to consider in planning for the database.

The curriculum map developed with the above considerations can be used to track individual programmes and courses/modules as well as individual lessons, and can also track multiple programmes including different versions of the same programme ( Kang, 2012). For example, the original curriculum information for many programmes are retained in the database and each time the programme or even a single module/course undergoes a revision, the database is updated and is able to display the original curriculum and the updated curriculum. In the example that appears in Figure 2, 3, 4, the table displays the Pharmacy 2012 and 2017 curricula. This capability allows the database to track any changes and to also identify which cohort of students have been exposed to which revision of the curriculum. Additional examples on this is displayed in Figure 3. At the IMU, the curriculum map is known as the curricula database and it involves all theundergraduate programmes (12) in the university and tracks 109 separate database fields, of which 58 of them are curricula descriptors. The database was also contextualised to the IMU’s and the Malaysian Accreditation Board requirements such as the Code of Practice for Programme Accreditation (COPPA) for the cognitive, affective and psychomotor domains and soft skills ( Malaysian Qualifications Agency, 2007).

It is capable of tracking any keyword, or term and map it to outcomes, teaching learning methods, curricula content and assessment tools. Other than blueprinting assessment tools to learning outcomes, the database allows for blueprinting of Malaysian Qualification Agency accreditation COPPA domains to components in the curricula for each programme.Reference materials including books, websites and e-learning links can also be included and tracked across the various programmes.

#### The IMU Experience:

The route IMU had taken was for a rapid development of the database application based on the FileMaker platform ( www.filemaker.com). This platform allows for the user to develop customised solutions with either available templates or using a ground up approach in creating the database. Health professions (education) faculty’s expertise does not usually include application development, and typically, most staff have a general understanding of spreadsheets, presentation tools, and word processing. The decision at that time in early development of the database was to look for a rapid development database application system which worked both on the Windows and Macintosh platforms, which could be grown as the need arises and uses a graphical interface. The development of the database would be conducted by a single faculty whose requirement were originally driven by the needs to track the various teaching and learning activities for accreditation purposes and later for student revision and faculty tracking of the curriculum. These activities should also be searchable using key words or via natural language. An additional advantage was that the search results can be arranged, filtered or sorted and this feature was provided by the FileMaker platform.

Additional requirements were that it is a stand-alone application which could be assessed from the web browser (for users) and that the database could also grow in complexity with additional functions, graphics, search and analytics functions, and integration to the Learning Management System. This database/curriculum map also provided links to the location of the teaching and learning material residing on the LMS and this includes resources for formative assessments.

### Phase 3: Database Mining and Graphical Output

The curricula database had embedded analytics functions where the search results can be analysed and sorted using any function or fields to give comprehensive details ( Table 3). It addresses the issue of making the curricula transparent to the students, faculty and administrators. It can generate reports for mapping of the curricula to outcome descriptors and this leverages on the capabilities of a relational database. This database enabled individual tracking of fields within a programme as well as the ability to track keywords or topics which may be taught across programmes (eg. identifying how ethics and professionalism is delivered by different programmes). Such mapping helps staff and administrators during the accreditation and curriculum review processes, and since access of curricula map is through the cloud, everyone in the university including students can view the map. Additionally, the relational structure of the database allows staff from different programmes to look for opportunities to conduct common learning as well as inter-professional learning, thus maximising the use of scarce resources. Figures 2, 3, 4 illustrates an example where the database was mined to show a mapping between a lesson/topic with learning domains and programme outcomes. In Figure 3, the top diagram illustrates a typical view of the database, which the dropdown box allows a selection of various programmes, including different revisions of the programme. Here, Biomedical Science, Pharmaceutical Chemistry, Pharmacy, Dentistry and Medical Biotechnology have various versions of their programmes which can be tracked. Selection of the Programme will then allow the user to select the list of modules available (bottom left) and following that, the user can sort the list of teaching and learning activities into a table as shown in the bottom right. In Figure 4, the top diagram illustrates how another search can be initiated. From the topic or other appropriate fields, the user can search for a term. In this case, ‘cancer treatment’ was the search term in which the database matches it for where this appears throughout all the programmes and had turned out a table of matches in Figure 4.

**Table 3.** Example of curricula mapping results obtained with analytics

	Using Data from	Search Through/For	Analytics applied	Example
1	Programme/Semester/Module	Learning Activities	Sort the data into delivery types encompassing soft skills, cognitive, affective and psychomotor domains	Searching for where ophthalmology is taught in Medicine and how the various domains are used to achieve the outcomes.
2	Outcome domains	Undergraduate programmes	Identifying which programmes or modules covers entrepreneurship as a subject	Searching for where “Entrepreneurship” is delivered for any programme in the university and if inter-professional learning activities are possible.
3	Programme/Semester/Module	Accreditation requirements in soft skills domains	Sorting the delivery by module, semester or delivery type	Searching through the Nursing programme to identify where “thinking outside the box” takes place in the programme
4	Assessment blueprint	Tools used for summative assessment	Sort the data into programmes, modules or semesters which then can identify support of reflective writing for the students and staff	Searching for reflection as a summative assessment in the curriculum and identifying where teaching and learning resources are needed to support both staff and students.
5	Support material and references recommended for student use	Usage by different programmes	Analysis of the resources used can help the chief librarian to manage the inventories.	Searching for textbooks by a certain author or title and if it is used in any programme.
6	Programme outcomes	Mapping various outcomes from different programmes to the accreditation body outcome domains	Analysis of the delivery at particular time points	Identification of potential of inter-professional learning across different cohorts to achieve similar outcomes.

#### The IMU experience:

To manage the different categories of users, the decision taken by the developer was to segregate users to the following categories:

• Admin users - super user rights; able to add, delete, edit records and database tables and the entity relationship diagram of the database,
• Curricula manager users - these users have editing rights and are able to add, delete and edit records in the database,
• Non-editing users - these users are the bulk of the users and comprise of students and faculty. They have no editing rights, and have access to the entire curricula, with viewing and searching rights only.

The curricula database was introduced to both students and faculty and both parties had provided feedback to the development of the database. Both groups are provided training on how to use the database and generally both groups need to understand how to use the database to search through the curriculum and how they can apply the sort and analytics functions for the data they obtained through the search activity. Another sub-group of curriculum manager (also known as programme directors), have the duty and responsibility to update the database whenever approved modifications are authorised by Senate. These group of programme directors are allowed editing rights, whereas other users have only viewing rights and will not be able to change anything on the database.

The database undergoes a backup process every day and built into the structure of the database is who had accessed, made changes and when the changes were made. This built in log file assists in monitoring and accountability for any changes that are expressed in the curriculum database.

Training is provided for all staff and students on how to use the database. Additionally, the database has a built-in Frequently Asked Questions section where any user can refer and learn how to use the database.

#### The challenges faced:

(1) Structure of the database: The structure of the database is an important decision as the wrong structure will result in the inability for the database to search, analyse and generate reports and graphical analysis. It is recommended that in developing the database structure and the entity relationship diagram (ERD), that personnel who are fluent in developing database structures are involved and are included in part of the team.

(2) Faculty and staff buy-in: A curriculum map is a living document and will need to be updated on a periodic basis to reflect the currency of the curriculum. These are challenges for faculty and the curriculum administrator. One solution to solve this issue is to provide Word/Excel templates with the curriculum details so as to aid the automatic import of the information into the database. Currency of the curriculum data can be tracked by four fields in the database which identifies the person who enters the data, the person who modifies the data, was modified.This information is supported with a log file which contains the history of changes.

(3) Standardised terminology and nomenclature: This is an important issue to consider, especially when multiple programmes from different disciplines are using the curriculum database. A common example is the use of the term lecture and plenary interchangeably. To resolve this issue, an extensive frequently asked questions (FAQ) section on the database includes the list of terminology accepted and used in the database.

## Lessons Learnt

The process of developing a bespoke curriculum map using a database is an involved, iterative, and consultative process. The process of mapping should be started with a single programme, preferably one with many common educational elements across the university. One alternatively can construct the database one each for individual programmes, however, such a decision does not exploit the capabilities of the curriculum database to map through the entire curricula.

Other considerations are the accessibility and security access for users. Depending on the security and privacy level of the university and policies, the access to the database may be limited to users accessing it from specific IP addresses (campus limited). The curricula map data generally belongs to the university and there must be security processes for those accessing it. Privileges for those who are viewing the data must be different from those who are updating or involved in data entry to distinguish between the two tasks and to prevent accidental deletion and modification. There should be a built-in log file to track what are the changes made and the individuals who have made the changes it. Another important matter is the how the curriculum map would look for the user on the computer and on mobile devices. The screen real estate for these devices are different and the application should dynamically recognise the access of such devices and display appropriate information on demand. Finally, the curricula database project should be self-sustaining, that is if the programmer/developer is no longer available, the database can be run with minimal human intervention.

## Moving Forward

Curriculum mapping database with built-in analytics is an important tool to assist with curriculum management, resource tracking, and curriculum review processes. Ultimately, curriculum mapping is used to assist teaching and learning as well as preparation of learning plans by the students. Integration between the database with the learning management system (LMS) can enable the learners to be responsible for their own learning plans. For curriculum and university administrators, the database can be modified to provide a dashboard to provide a macro view of the curriculum for resource planning and administration.

Furthermore, integration of artificial intelligence (AI) to track student usage of the database can provide insight into the user engagement which can but help improve the outcomes on how the map can be used to improve outcomes.

## Conclusion

The curricula database is useful and powerful tool in mapping, tracking and identifying curricula data and events. This becomes even more powerful with the addition of analytics, which enables the sorting of the queries.

## Take Home Messages

• Planning the Curriculum Database Structure
• Database Application Development
• Development of the Application
• Database Mining and Graphical Output

## Notes On Contributors

Yew-Beng Kang is an Associate Professor and the Associate Dean E-Learning, International Medical University, Kuala Lumpur, Malaysia. ORCID: https://orcid.org/0000-0001-6733-8880

Vishna Devi V. Nadarajah is a Professor and the Pro Vice-Chancellor of Education, International Medical University, Kuala Lumpur, Malaysia. ORCID: https://orcid.org/0000-0002-7126-7189
