# Supplementary material for: Building a database for curriculum mapping and analytics
Source: MedEdPublish (2016). 2019 Aug 19;8:38. Originally published 2019 Mar 4. [Version 2] doi: 10.15694/mep.2019.000038.2 (PMC10712633; doi:10.15694/mep.2019.000038.2)
Supplement: Peer review history

# Reviewer report: Reviewer response for version 2

**DOI:** 10.21956/mep.20240.r31376

**Published:** 2019-11-23

**Authors:** P Ravi Shankar

**Affiliations:** 1 American International Medical University

This review has been migrated. The reviewer awarded 4 stars out of 5 I had enjoyed reading the original version of this paper and the revised version submitted by the authors. I mainly got interested in a curriculum database through a series of interaction with accreditators and accrediting agencies in the Caribbean. Most medical programs have a set of defined outcomes and one of the questions commonly asked is where a particular outcome is addressed in the curriculum and the teaching-learning methods. There is now a number of commercially available curriculum mapping programs in North America and we had examined a number of them for possible application at a new Caribbean medical school. The International Medical University in Malaysia offers a number of educational programs in health professions at various levels opening up opportunities for interprofessional learning and collaboration. Also many of the commercially available programs are primarily focused around North American requirements and the standards of North American accrediting bodies. There is bound to be differences in emphasis and in standards in Asia so I find the authors approach to be important and interesting. This well-written manuscript details the processes involved in developing a curriculum database. I found table 3 to be very interesting as it details examples of mapping results which can be obtained using the database. One of the challenges which I have faced is the absence of an educational technologist in the team which hampers our ability to develop different educational software and programs. A curriculum mapping software integrated with the overall admissions, learning management system and academic operations system can be a powerful solution and can assist faculty, administrators and students in a variety of ways. I found some of the technical terms related to databases required a second more careful reading but my experience with a commercial curriculum mapping software has made me more aware of the capabilities of the IMU database. I congratulate the authors on their initiatives. All medical and other health system educators will find this article to be of interest. In line with developing video and other capabilities of the e-journal I am of the opinion that a brief video demonstration by the authors of the mapping system will also be of interest.

Reviewer Expertise:

NA

No decision status is available

# Reviewer report: Reviewer response for version 2

**DOI:** 10.21956/mep.20240.r31379

**Published:** 2019-10-25

**Authors:** Barbara Jennings

**Affiliations:** 1 Norwich Medical School

This review has been migrated. The reviewer awarded 4 stars out of 5 As with the first version of the article, I think this is a well-written resource review and a useful account of an important aspect of curriculum management. The authors have cited several key papers about course mapping and the use of analytics that will be of interest to anyone responsible for the design, development, and quality assurance of curricula. The open peer reviews linked to both versions of this article also make interesting reading – because the reviewer comments and questions demonstrate the challenges faced by academic teams in curriculum management.In the IMU-Experience section of the Methods, the authors compare and contrast the use of spreadsheets and databases for curriculum mapping and clearly justify the use of a relational database. I appreciated the clear explanations of generic principles of project management for curriculum design, coupled with the specific considerations for the authors’ project. Thank you for explaining the Integration options for other systems and the ability to import and export data.The take home messages section is a bit brief; more of a list of headings- perhaps the authors could have elaborated on each, as a brief summary of guidelines that readers could consider? Nevertheless, I think there are important messages in this article for course conveners in particular.

Reviewer Expertise:

NA

No decision status is available

# Reviewer report: Reviewer response for version 2

**DOI:** 10.21956/mep.20240.r31377

**Published:** 2019-09-28

**Authors:** sathyanarayanan varadarajan

**Affiliations:** 1 SRM Medical College, SRM University

This review has been migrated. The reviewer awarded 4 stars out of 5 Thanks to the editorial team for inviting me to review this paper..This interesting paper reports on the implementation of a university-wide curricula database which includes multiple undergraduate and postgraduate programmes (medicine, dentistry, pharmacy) after considering the needs of everyone who will have access to the database. The authors have clearly outlined the steps to design the curricula database, the development of the framework of the database and the analytics, challenges in implementation, the results that can be obtained from such a database and the lessons learnt with appropriate tables and figures. The authors also included specific sections on their institutional experience which expands the decisions, options and key ground rules set to develop the database.They’ve also highlighted many unique features of their tool, the challenges faced and the ways of moving forward. The authors conclude by stating that the curricula database is useful and powerful tool which becomes even more powerful with the addition of analytics, which enables the sorting of the queries.Curriculum mapping database with built-in analytics tool will definitely help in assisting with curriculum management, resource tracking, and curriculum review processes. So this article will be helpful to all the medical educators and administrators who are involved in designing, developing, planning, managing and evaluating the curriculum.

Reviewer Expertise:

NA

No decision status is available

# Reviewer report: Reviewer response for version 2

**DOI:** 10.21956/mep.20240.r31378

**Published:** 2019-08-23

**Authors:** Mohamed Al-Eraky

**Affiliations:** 1 University of Dammam

This review has been migrated. The reviewer awarded 2 stars out of 5 Thank you for your interesting paper that explains the methods used to develop your database for course mapping. You may need to elaborate more on how did you make the constructive alignment. To me, Table 1 was informative, but not detailed enough. Teaching activities should not only indicate the method or strategies (e.g. small/large group or teacher/student-centered), but you need to provide a full description on the activity, including: the task, how students are expected to perform it, time allocated, mode of submission, and how teachers would assess their performance with respect to specific learning outcome. Also, I see the language of the paper is more suitable for technical IT readers, as demonstrated in Table 2 and almost all figures. While the paper is expected to address mainly the educational aspects and features of the database that would be of more interest to teachers, educational leaders, curriculum committee members and even students. I am sure that your product is innovative, yet it could have been presented with an educational perspective to suit medical education readership in MedEdPublish. Good luck.

Reviewer Expertise:

NA

No decision status is available

# Reviewer report: Reviewer response for version 1

**DOI:** 10.21956/mep.19120.r27591

**Published:** 2019-03-24

**Authors:** Gominda Ponnamperuma

**Affiliations:** 1 Faculty of Medicine, University of Colombo

This review has been migrated. The reviewer awarded 4 stars out of 5 This should be a very useful paper for anyone who wishes to map a health sciences curriculum electronically. This is something which is very difficult to explain in a paper such as this. However, to the credit of the authors, they have made a commendable effort. I would like to know more about the different options that were available to the authors at the different stages of the development of this database, and the pros and cons of selecting a given option over the others. Such a discussion, particularly with regard to the layout and the functionalities (the function buttons), would definitely assist any prospective developer of a similar database.

Reviewer Expertise:

NA

No decision status is available

# Reviewer report: Reviewer response for version 1

**DOI:** 10.21956/mep.19120.r27589

**Published:** 2019-03-11

**Authors:** Barbara Jennings

**Affiliations:** 1 Norwich Medical School

This review has been migrated. The reviewer awarded 3 stars out of 5 I think this is an interesting and well-written paper about an important aspect of curriculum management. It will be of interest to anyone responsible for the design, development, and quality assurance of curricula. Keeping track of learning objectives; documenting the intersection and integration of learning objectives; and being clear about their purpose in the education of a competent graduate are key concerns for all teachers and course conveners. But designing systems to facilitate that tends to rest with data managers within departments, or at an institutional level. This is a well-written resource review. I appreciate this type of article for allowing departments to share good practice and exchange ideas and experiences of course management. The bibliography will be helpful to readers who need to review the literature about mapping and course analytics. However, the software and system described here was driven by the quality management needs of a specific higher education institution, which probably limits the generalisability of this paper.I noted the sage advice about the importance of creating logical and intuitive systems. It is indeed important for an organisation to develop tools and infrastructure that do not require extensive historical knowledge to operate. I would like to know about any feedback that the authors have had from other faculty members since the implementation of the new database & analytics system? I also wondered about whether additional external software could be integrated with the database? For example, if there is a valuable new formative exam bank that students could use to support their learning – could it be linked to this database in a logical way?

Reviewer Expertise:

NA

No decision status is available

# Reviewer report: Reviewer response for version 1

**DOI:** 10.21956/mep.19120.r27590

**Published:** 2019-03-07

**Authors:** Ken Masters

**Affiliations:** 1 Sultan Qaboos University

This review has been migrated. The reviewer awarded 2 stars out of 5 A useful description of the process involved in developing a curriculum map, and, without going into too much technical jargon, the authors have been able to explain technicalities involved.An important issue not mentioned in the article, however, is the impact of changes to the curriculum. The article does refer briefly to updating, and log records, but then does not explain what happens to the old data. This is crucial when faculty and students come to actually using the map: • For Faculty: In 2019, I teach a course in 3rd year that relies on Anatomical knowledge, and I want to know what these students were taught in their years before. If I look at the curriculum map, I can see what is being taught to first year students and second year students in 2019. That is useful, but does not serve my purposes, because I know there were changes some time last year. I need to know what this cohort of students was taught in 2nd year in 2018, and what they were taught in 1st year in 2017. Also, I have three students who repeated courses in their 2nd year, one of whom had a leave of absence for one year, and five students who repeated courses in their 1st year, so I also need to know what those students were taught when they were in those years. Can I find this information in the map?• For Students: similarly, students in their 3rd year want to check back to see what they were taught when they were in 1st and 2nd year, not what is being taught in 1st and 2nd year now. Can they do this, and get access to those materials?Without this, the map is useful for administrators and the institution for administrative and accreditation purposes, but the benefit to Faculty and Students is marginal. If the curriculum map can accommodate these (or some of these), it would be useful if the authors could describe this, and how it does this (including a screenshot or two). If it is not able, then the authors should describe it as a limitation (that the map gives current information only, and has no “memory” of past classes and materials; it may be “living”, but it is a static snap shot of the “now”), why it was not built into the system, who decided against it, and also give some idea of whether this is being worked on for future developments of the map. Small issues:• The information under the sub-heading “Database Application Development” tends to repeat what is in Figure 2. I think it is enough to refer simply to the steps in Figure 2 without repeating the information.• Figure 2 should more correctly be labelled Table 1, and the current Table 1 should be Table 2.• “are built-into the” should be “are built into the” (and a few other language errors should also be addressed).• This sentence appears to have errors and needs to be corrected: “Currency of the curriculum data can be tracked by four fields in the database which identifies the person who enters the data, the person who modifies the data,was modified.”

Reviewer Expertise:

NA

No decision status is available

# Reviewer report: Reviewer response for version 1

**DOI:** 10.21956/mep.19120.r27588

**Published:** 2019-03-04

**Authors:** P Ravi Shankar

**Affiliations:** 1 American International Medical University

This review has been migrated. The reviewer awarded 4 stars out of 5 The authors in their interesting manuscript describe the development of a bespoke curriculum management system at the International Medical University in Malaysia. A brief description of how the system was developed and how it is being used is provided. I would be interested in knowing about factors to consider at a technical level while developing the system. Does this require a detailed knowledge of database management and programming? At our medical school we are considering using a commercial curriculum management system (CMS). The authors also mention that the CMS should be linked to the student management and the learning management system. What systems do they use for these purposes at IMU? Figure 2 will be of interest to the readers. It will be helpful if the authors can describe the issues mentioned in this table in greater detail. With regard to figure 3 the lesson outcomes have not been properly written using the standard terminology used for learning outcomes. There are a few language errors at a few places which can be corrected. The paper would be of interest to all medical educators especially considering the fact that CMS is becoming an important component of curricular management and review.

Reviewer Expertise:

NA

No decision status is available
